# Effects of a sleep program on sleep, performance and health in university students: Study protocol for a randomised controlled trial

**DOI:** 10.1371/journal.pone.0361815

**Published:** 2026-10-30

**Authors:** Eivind Andersen, Fredrik Lie Haugen, Heidi Lindsverk, Oda Eline Aasland, Anne Sæther, Bjørn Bjorvatn

**Affiliations:** 1 Faculty of Humanities, Sports and Educational Science, University of South-Eastern Norway, Tonsberg, Norway; 2 Faculty of Humanities, Sports and Educational Science, University of South-Eastern Norway, Tonsberg, Norway; 3 Faculty of Humanities, Sports and Educational Science, University of South-Eastern Norway, Tonsberg, Norway; 4 Faculty of Humanities, Sports and Educational Science, University of South-Eastern Norway, Tonsberg, Norway; 5 Faculty of Humanities, Sports and Educational Science, University of South-Eastern Norway, Tonsberg, Norway; 6 Department of Global Public Health and Primary Care, University of Bergen, Bergen, Norway; Hong Kong Metropolitan University, HONG KONG

## Abstract

**Background:**

Insomnia and poor sleep are highly prevalent among university students and are linked to impaired mental and physical health, reduced cognitive functioning, and lower academic performance. Existing interventions for insomnia include cognitive behavioural therapy for insomnia (CBT-I), mindfulness, and sleep hygiene. CBT-I is widely recognised as effective but remains difficult to access for the general population and lack scalability, long-term follow-up, and feasibility in non-clinical settings. Pragmatic, group-based approaches that can be delivered within the university and are tailored to help students develop self-endorsed routines and competencies for better sleep, creating sustainable changes that extend beyond the program period, is needed.

**Methods:**

This study is a pragmatic, two-arm randomised controlled trial evaluating the effectiveness of a group-based sleep program for university students with insomnia symptoms. A total of 118 full-time students aged 18–30 meeting diagnostic criteria for chronic insomnia disorder will be randomized (1:1) to either the intervention or a wait-list control group. The intervention comprises 12 weekly 90-minute group sessions followed by nine monthly follow-up meetings, facilitated by trained coaches. The program integrates core elements of CBT-I with motivational and coping strategies, delivered in an interactive and peer-supported format. The primary outcome is change in insomnia severity measured by the Bergen Insomnia Scale. Secondary outcomes include subjective and objective sleep measures, mental health, cognitive performance, academic attainment, health behaviours, physical fitness, and biological markers. Assessments will be conducted at baseline, post-program (12 weeks), and 12 months. A mixed methods process evaluation will assess fidelity, acceptability, mechanisms of impact, and contextual influences.

**Discussion:**

This trial will be among the first pragmatic evaluations of a structured, group-based sleep intervention tailored to university students with insomnia. Key strengths include the randomised controlled design, comprehensive outcome assessment, 12-month follow-up, and embedded process evaluation. If effective, the program could provide a feasible, scalable, and cost-effective model to improve sleep and therein health, and academic performance in young adults in higher education.

## Background

Sleep is a fundamental physiological process essential for health, well-being, and daily functioning. Despite this, insufficient and poor-quality sleep is highly prevalent among university students. Large-scale surveys consistently show that students sleep less than recommended, with many experiencing irregular sleep patterns and difficulty maintaining healthy routines [1–5]. Contributing factors include academic demands, irregular study schedules, evening use of digital devices, caffeine use, part-time employment, and social activities that delay bedtime [4]. In addition, stress, financial strain, and mental health problems such as anxiety and depression are common in this age group and strongly linked to sleep difficulties [2,3]. Together, these factors create a context where disrupted and insufficient sleep has become the norm rather than the exception among students. Insufficient sleep in this population has wide-ranging negative consequences. Insufficient sleep is associated with impaired immune function, metabolic and cardiovascular risk factors, and increased vulnerability to illness [6–9]. Equally important are the mental health effects where short and irregular sleep is linked to heightened stress, symptoms of anxiety and depression, and reduced emotional regulation [10]. Cognitive consequences include impaired attention, learning, and memory consolidation, all of which are central to academic achievement [11]. In contrast, adequate and good-quality sleep contributes to physical recovery, emotional stability, effective learning, and improved performance as a student [12,13]. Promoting better sleep in young adults is therefore not only a matter of physical and mental health, but also a key factor in academic success and overall quality of life.

Several interventions have been developed to improve sleep in student populations, often drawing on cognitive behavioural therapy for insomnia (CBT-I), mindfulness or sleep hygiene education [14]. Systematic reviews and meta-analyses indicate that such interventions can produce moderate-to-large short-term improvements in sleep quality and insomnia symptoms, with some additional benefits for anxiety and depression [10,15–17]. However, existing research in this field has important limitations. Many studies rely on small samples and are carried out under controlled rather than real-world conditions, which raises questions about scalability and feasibility in typical university settings. Moreover, interventions are often brief, include a non-active control group, lack objective measurement of sleep, has a narrow intervention focus (i.e., only sleep hygiene, mindfulness), or lack active engagement strategies, limiting their impact. Furthermore, long-term follow-up is rare, leaving uncertainty about whether improvements in sleep can be sustained over time. Overall, meta-analyses consistently indicate a substantial risk of bias across the included studies [10,16,17].

The present study seeks to address many of these gaps by implementing a comprehensive pragmatic, long-term, interactive group-based sleep program tailored to university students. Here, ‘pragmatic’ refers to the trial’s real-world delivery conditions (non-specialist coaches, typical university setting, broad eligibility, intention-to-treat analysis), consistent with the PRECIS-2 framework, rather than to the intensity of the intervention itself. The program consists of 12 weekly interactive sessions followed by monthly follow-ups for nine months, combining evidence-based elements from CBT-I with motivational and stress-coping strategies, and supported by peer interaction. The program is designed to be feasible within a university context, emphasizing active participation, reflection, and the development of sustainable behaviour change.

An increased recognition of sleep as a fundamental determinant of both physical and mental health, alongside the well-documented adverse effects of sleep difficulties on psychological well-being, highlights the pressing need for effective interventions aimed at enhancing sleep quality. This paper describes the protocol for a randomised controlled trial (RCT) evaluating the effectiveness of the program compared to a waiting list control group, who will be offered the program after the 12-month follow-up. The primary aim is to determine whether the program improves sleep outcomes among students with insomnia symptoms. Secondary aims are to assess potential effects on mental and physical health, cognitive and academic performance, physical fitness and physical activity and diet, and overall quality of life. A process evaluation will be conducted to explore implementation and participant experiences.

## Methods

### Study design

This study is a pragmatic two-arm randomised controlled trial to assess the short- and long-term effects of a sleep program on students with insomnia in a Norwegian University. In total, 118 participants will be recruited. Fig 1 summarises the study design using the CONSORT template. The study has been approved by the Norwegian Regional Committees for Medical and Health Research Ethics (reference number 950880) and will be reported in accordance with CONSORT with intention-to-treat analyses. The trial is registered in ISRCTN (17008034).

**Fig 1:**
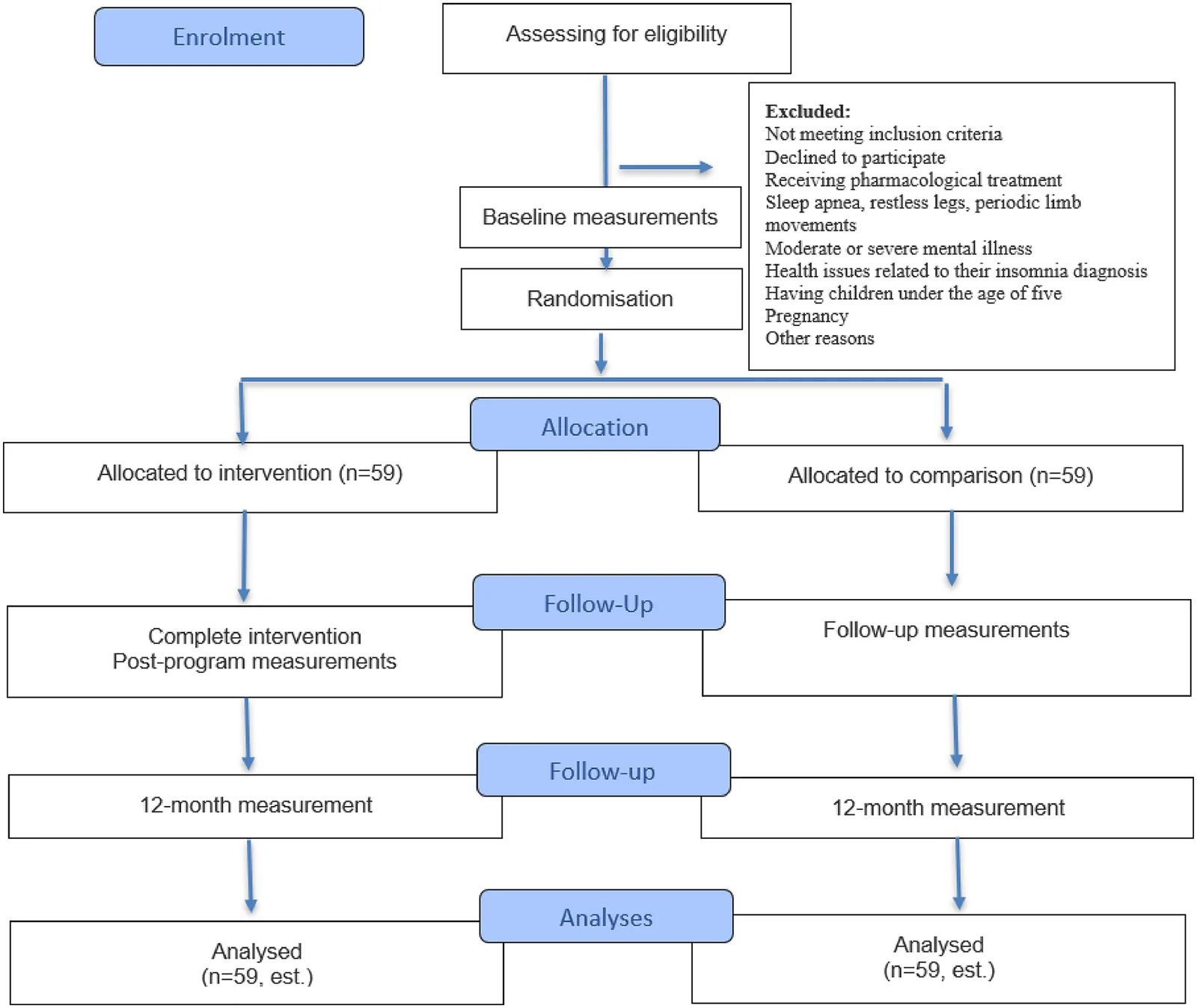
Shows the flow of participants through the trial.

### Participants

The participants are fulltime university students with insomnia symptoms. Participants with specific health issues that could possibly explain the insomnia symptoms will be excluded. To better be able to create homogenic groups, only young adults are targeted.

Inclusion criteria:

- Meet the criteria for chronic insomnia disorder [18] based on the Bergen insomnia scale (BIS) [19]. The BIS includes six items, with chronic insomnia defined as scoring ≥3 on at least one of the first three items and ≥3 on at least one of the last two items.
- Student at the University of South-Eastern Norway (USN), with a minimum of two years remaining in your studies
- Aged 18–30 years
- Understand and speak a Scandinavian language
- Consent to randomisation

Exclusion criteria:

- Receiving pharmacological or non- pharmacological treatment for sleep problems
- Sleep apnea, restless legs or periodic limb movements, because this may suggest that other sleep disorders may explain the insomnia symptoms
- Moderate or severe health issues related to their insomnia diagnosis
- Unstable or severe medical/neurological conditions that substantially affect sleep
- Moderate or severe mental illness
- Drug or alcohol abuse
- Current nightshift or rotating-shift work (≥1 night shift per week within the past month or anticipated during the trial)
- Having children
- Pregnancy

### Intervention program

The intervention is a pragmatic group-based sleep program designed to support university students with insomnia in improving their sleep and maintaining long-term change. The program is delivered over 12 weekly 90-minute sessions, followed by monthly group meetings for nine additional months (i.e., one year from baseline assessment).

Groups will consist of approximately 20 students, each facilitated by a trained coach. Sessions are interactive, discussion-based, and experiential rather than didactic. The style of delivery emphasizes creating an autonomy-supportive and collaborative climate, where participants are encouraged to reflect, set personal goals, share experiences, and learn from peers. Coaches provide a rationale for behaviour change, help participants identify their own strategies, and foster a supportive group environment that values effort and progress as much as outcomes.

The program is grounded in evidence-based behaviour change principles. Cognitive Behavioral Therapy for Insomnia (CBT-I; focusing on identifying and modifying maladaptive thoughts and behaviours related to sleep), Self-Determination Theory (SDT; supporting autonomy, competence, and relatedness) and Stress and Coping Theory (SCT; focusing on cognitive appraisal of stressors and the use of coping strategies). These theories guide the program’s focus on building intrinsic motivation for sleep-promoting behaviours, enhancing self-regulation skills, and providing relapse prevention strategies.

The 12 core sessions each have a thematic focus*:

1. What is sleep? Sleep and health: Understanding sleep mechanisms and circadian rhythms, and the importance of sleep for physical and mental health, cognitive function, and performance.
2. Sleep diary and goal setting: Establishing self-monitoring and individualized goal plans.
3. Sleep hygiene: Identifying and adopting practical routines.
4. Different sleep disorders, and causes of sleep problems: Exploring biological, psychological, and social contributors.
5. Physical activity, physical fitness and diet: Exploring how these influences sleep.
6. Wider influences: The role of stress, financial concerns, social life, anxiety, and depression.
7. Sleep restriction: Considered to be the most effective component of CBT-I. Increasing the homeostatic drive for sleep. Participants discuss potential benefits and challenges and reflect on whether aspects of sleep scheduling could be relevant.
8. Stimulus control: Aims to correct maladaptive sleep behaviours and strengthen the bed–sleep association. Patients are instructed to use the bed only for sleep (sexual activity permitted), and to leave the bedroom if unable to fall asleep within 15–30 min, returning only when sleepy. Wake-up time is fixed and should not be adjusted according to actual sleep duration. Participants discuss potential benefits and challenges and reflect on whether aspects of stimulus control could be relevant.
9. Relaxation techniques: Practicing breathing, mindfulness, and muscle relaxation.
10. Light therapy, melatonin and other possible complementary approaches: Evidence-based use and limitations.
11. Cognitive techniques (inspired by CBT-I): Introducing selected strategies from cognitive therapy. Participants reflect on common unhelpful thoughts about sleep and practice simple re-framing exercises. Coaches facilitate reflection but do not provide clinical therapy, students in need of structured CBT will be referred to professional services.
12. Summary and looking ahead: Reviewing key lessons from the program, consolidating individual progress, and planning for the maintenance phase. Participants reflect on what strategies worked best for them, identify potential barriers moving forward, and set personal action plans.

* The sessions provide participants with tools for self-reflection and peer discussion. Coaches will be trained to facilitate the exercises but will not act as therapists. Participants who require more comprehensive therapy will be encouraged to seek professional health services.

Each session combines short inputs from the coach with individual and group-based exercises. Typical tasks include reflecting on positive and negative effects of sleep, identifying personal causes of sleep difficulties, generating solutions, and discussing barriers to change. Homework assignments between sessions include keeping a sleep diary, applying sleep hygiene strategies, practicing stimulus control or relaxation techniques, incorporating sleep restriction if applicable, and setting new weekly goals. Participants are encouraged to experiment, learn from setbacks, and refine their strategies over time. The self-help book *“Bedre søvn – en håndbok for deg som sover dårlig”* (“Better sleep. A handbook for you who sleep poorly”) [20] covers normal sleep and sleep regulation, how sleep problems are assessed, and describes different causes of poor sleep. The main focus of the book is CBT-I. The book serves as a core resource to complement group work.

Following the initial 12-week program, participants attend monthly follow-up meetings for nine months. These “light-touch” sessions focus on relapse prevention, reinforcing behavioural strategies, maintaining motivation, and providing peer support. They give participants the opportunity to reflect on challenges, share solutions, and celebrate progress.

In summary, the program builds on the following elements aimed to help students develop self-endorsed routines and competencies for better sleep, creating sustainable changes that extend beyond the program period:

- Drawing explicitly on motivational theories to foster internalized, personally meaningful reasons for change.
- Supporting the development of self-regulation strategies (e.g., goal setting, self-monitoring, implementation intentions).
- Embedding behaviour change tools in enjoyable, interactive sessions designed to sustain engagement.
- Providing relapse prevention techniques and long-term peer support.
- Incorporating evidence-based components from CBT-I, including stimulus control, sleep restriction, and cognitive restructuring.

The programme content and structure were developed by the research team, grounded in the CBT-I, Self-Determination Theory, and Stress and Coping Theory frameworks described above. Prior to the start of the trial, the programme will be piloted with a group of students with lived experiences of sleep difficulties, and refined based on their feedback.

### Comparison group

As a waiting list control group, the comparison group will be placed on a wait list to be offered a guaranteed place on the program after their 12-month follow-up measurements are completed. In addition, all participants (both intervention and comparison group) will receive a brief sleep-hygiene leaflet following the baseline measurement and prior to randomisation (Table 1). The leaflet provides general information only and does not include core programme components. To minimise contamination, participants in the comparison group will be asked not to start other structured sleep treatments during the trial, any co-interventions will be recorded.

**Table 1:** Sleep hygiene advice [21].

• Avoid caffeinated drinks during the last hours before bedtime (coffee, tea, cola, energy drinks) • Avoid going to bed hungry, but do not consume a heavy meal or alcohol before bedtime • Keep your bedroom quiet, relaxing, and at a cool temperature • Turn off electronic devices at least 30 minutes before bedtime • Exercise regularly and maintain a healthy diet, but do not exercise during the last hours before bedtime • Go to bed and get up at the same time every day

## Data collection

### Recruitment

The participants are students recruited from USN, primarily campus Vestfold. Campus Vestfold has approximately 5000 students. Drawing on findings from the SHOT study, which reported a 30.5% prevalence of insomnia in a national survey of Norwegian university students [1], an estimated 1600 students at this campus could potentially be eligible for inclusion. Participants will be recruited from May 2027 onwards and will continue until we reach 118 eligible students.

We will use a multi-channel recruitment strategy: invitations via university e-mail, digital screens across campus, posts on the university intranet and official social-media accounts, and brief in-person announcements in lectures and at student events. In addition, we will collaborate with student unions, student health/counselling services, and programme administrators to extend reach.

Students will be able to register their interest online through a provided link (developed for the study and linked to the study database). The research team will then phone all students who have registered an interest in taking part in the programme. The researchers will discuss the study and conduct a screening for eligibility. Eligible participants will be sent a confirmation e-mail, including the participant-information sheet, a consent form and an appointment to attend an information meeting at the University. At this information meeting, researchers will explain the study procedures and inclusion criteria, and take students written informed consent for taking part in the study. Those who agree to take part in the trial will be asked to indicate in writing whether they are willing to provide optional blood samples. Although blood sampling in the present project is voluntary, prior research suggests that the majority of participants are willing to provide blood samples, and this is therefore not expected to meaningfully affect data completeness or the quality of the biological data [22].

Participants can leave the study at any time for any reason and without consequences. Intervention group participants who drop out from the program will still be invited to attend follow-up measurement sessions as part of the trial. If participants wish to fully withdraw from the study, their reason for leaving the study will be obtained via a structured phone interview, where possible. All participants will be offered a short feedback report after the 12-month measures which summarises their changes on key outcomes over the course of the trial.

### Randomisation

After the baseline assessments, the participants will be randomly assigned to either the intervention group or the comparison group. We will be using an individually randomised design because higher sample size and costs associated with a cluster randomised design are unwarranted as contamination risk is judged to be low given the intensive, in-person, group-based nature of delivery, which is not easily transferable between participants [23]. Nonetheless, use of external sleep-related resources or treatments will be recorded at each timepoint in both arms (see process evaluation). If non-trivial co-intervention or contamination is identified, sensitivity analyses excluding or adjusting for affected participants will be conducted. Participants will be randomly allocated to the intervention group or the waiting list comparison group in a 1:1 ratio. The method of randomised permuted blocks will be used, with random block lengths (4 or 6). The randomisation schedule will be generated by a computer program and stored within the University database, with access restricted to those responsible for maintenance of the randomisation system.

### Blinding

Because participants will be aware of their assigned intervention, blinding at the participant level is not feasible. Randomisation will take place after baseline assessments, ensuring that neither participants nor field staff are aware of group allocation at baseline. When baseline data have been collected, research staff will access the random allocation for each individual via a study web portal. Data processing and statistical analyses will be conducted by researchers blinded to group allocation.

## Procedures

Data will be collected at three time points: baseline (T0), immediately post-programme (12 weeks; T1), and 12 months after baseline (T2). Full details of the measures and the timing of each assessment are provided in Table 2. Assessment sessions will be scheduled on campus during class hours to maximise attendance. All measurements will be administered by trained research staff following standardised protocols and quality-assurance checks. Participants will complete a self-report questionnaire battery, a brief health screening, and standardised tests of cognitive function and physical fitness. Sufficient personnel will be present to assist participants who experience difficulties completing the questionnaires or tests. Validated and/or officially translated Norwegian-language versions exist for all questionnaires and outcome measures used in this study, and these versions will be used in the trial.

**Table 2:** Participant timeline: Schedule of enrolment, interventions, and assessments.

	TRIAL PERIOD			
	Enrolment		Post-randomisation	Close-out
TIMEPOINT	-T1 to T0	T0	T1 (12 week)	T2 (12 month)
***ENROLMENT***				
Eligibility screen (BIS, ISI, GSAQ, safety)	X			
Informed consent	X			
Baseline assessment session		X		
Randomisation (1:1)		X		
**INTERVENTION / COMPARATOR**				
Intervention: 12-week sleep programme		Start	End	
Comparator: Wait-list control + sleep-hygiene leaflet		Leaflet		Access to programme
**ASSESSMENTS**				
[Baseline variables and tests]				
Demographics (age, sex, education, employment hours, marital status)		X		
[Outcome variables and tests]				
Sleep (BIS, ISI, DBAS-16, SOL)		X	X	X
Mental health and wellbeing (PHQ-9, GAD-7, PSS-10, WHO-5, EQ-5D-5L)		X	X	X
Cognition and academic outcomes (PVT, executive function: 2-back or Stroop, exam results/credits)		X	X	X
Health behaviours and lifestyle (ActivPAL, IPAQ-SF, Diet & beverages, smoking/snus, AUDIT, DUDIT)		X	X	X
Somatic health and fitness (height, weight, BMI, waist circumference, blood pressure, body composition, VO_2max_)		X	X	X
Biomarkers (cortisol, hs-CRP, IL-6, TNF-α, glucose, HbA1c, C-peptide, insulin, lipids (TC, HDL, LDL, TG), homocysteine, Thyroid (TSH, Free T4), prolactin, BDNF, leptin og ghrelin)		X	X	X
[Other data variables and tests]				
Adherence, co-interventions			X	X
Adverse events / safety monitoring			X	X
Acceptability/usability and satisfaction			X	X
Learning Climate Questionnaire (LCQ-15)		X	X	X
Basic Psychological Need Satisfaction and Frustration Scale		X	X	X
Treatment Self-Regulation Questionnaire		X	X	X

BIS; Bergen insomnia scale, ISI; Insomnia severity index, GSAQ; Global sleep assessment questionnaire, DBAS-16; Dysfunctional Beliefs and Attitudes about Sleep, SOL; Sleep Onset Latency, PHQ-9; Patient health questionnaire, GAD-7; Generalized anxiety disorder, PSS-10; Perceived stress scale, WHO-5 Well-Being index, EQ-5D-5L; EuroQol 5 Dimensions – 5 Levels, IPAQ-SF; International physical activity questionnaire-short version, AUDIT; Alcohol use disorders identification test, DUDIT; Drug use disorders identification test, BMI; Body-mass index, VO_2maks_; maximal oxygen uptake, hs-CRP; High-sensitivity C-Reactive protein, IL-6; Interleukin-6, TNF-α; Tumor necrosis factor alpha, HbA1c; Hemoglobin A1c, TC; Total cholesterol, HDL; High density lipoprotein, LDL; Low density lipoprotein, TG; Triglyserides, TSH; Thyroid-Stimulating hormone, BDNF; Brain-Derived neurotrophic factor.

Anthropometry and blood sampling will be scheduled in the morning after an overnight fast, with participants asked to avoid vigorous exercise and caffeine for 12 hours beforehand. Retention will be supported with reminder messages and flexible make-up sessions. *The approach to missing data is described under Statistical analyses.* Adverse events related to assessments will be monitored and reported according to the study’s safety plan.

Primary outcome is change in sleep/insomnia severity, measured by the BIS questionnaire,Insomnia severity index (ISI) [24] is a key secondary sleep outcome. Additional secondary outcomes are (a) cognitive performance (brief, computerised tests); (b) academic attainment (examination results obtained from university records with consent); (c) mental health and wellbeing (e.g., anxiety, depressive symptoms, perceived stress, life satisfaction); (d) health behaviours (dietary habits, caffeine, alcohol use, and physical activity by questionnaire and accelerometery); (e) physical fitness (aerobic capacity); and (f) somatic health indicators (body composition; fat mass, lean mass; blood pressure; waist circumference; fasting blood biomarkers such as lipids and glucose), and lastly (g) C-reactive protein (CRP), cortisol, high-sensitivity interleukin-6 (IL-6), and the adipokines leptin and adiponectin, given their documented associations with sleep duration/regularity, autonomic balance, and cardiometabolic risk.

The **primary outcome** is insomnia severity, assessed with BIS, which is a validated instrument indexing the frequency and severity of core insomnia symptoms (sleep initiation and maintenance problems, early morning awakening, and non-restorative sleep). Items are rated for the past three months (0–7 days per item) and summed to a total score (0–42), with higher scores indicating a greater degree of insomnia.

**Secondary sleep outcomes** comprise sleep-related cognitions, and diary-based continuity/timing. ISI are rated during the last two weeks, and summed to a total score (0–28), with higher scores indicating a greater degree of insomnia symptoms.The Dysfunctional Beliefs and Attitudes about Sleep (DBAS-16) [25] captures maladaptive sleep-related cognitions, item means reflect the extent to which beliefs likely maintain insomnia. A seven-day sleep diary is used to derive standard continuity and timing metrics, including sleep onset latency (SOL), wake after sleep onset (WASO), time in bed (TIB), total sleep time (TST), sleep efficiency (SE = TST/TIB × 100), and sleep midpoint/bed- and rise-times.

**Mental health and wellbeing** are assessed with validated brief scales. Patient Health Questionnaire – 9 items (PHQ-9) [26] indexes depressive symptom severity and includes a suicidality item triggering the pre-specified safety response. Generalized Anxiety Disorder – 7 items (GAD-7) [27] indexes generalised anxiety severity. Perceived Stress Scale (PSS-10) [28] captures appraised stress over the past month. World Health Organization – Five Well-Being Index (WHO-5) [29] provides a positively framed measure of wellbeing/vitality. EuroQol 5 Dimensions – 5 Levels (EQ-5D-5L) [30] captures health-related quality of life across five domains and a 0–100 visual analogue scale.

**Cognitive function and academic attainment** target attention and executive control, domains sensitive to sleep disturbance. The Psychomotor Vigilance Task (PVT) [31] (5-min protocol) measures sustained attention/vigilance and outcomes include lapses (reaction time ≥500 ms), median reaction time, and false starts. For executive function, sites pre-specify one primary task: 2-back (letters) to index working-memory updating (outcomes: d′/accuracy and median RT) or a computerized Stroop task to index inhibitory control/interference resolution (outcomes: interference effect and accuracy). Tasks are delivered on standardized laptops with fixed scripts and practice to minimize learning effects, time-of-day is recorded for covariate use. Academic attainment is obtained, with consent, from university records for periods overlapping post-programme and 12-month follow-up, extraction is conducted by staff blinded to allocation.

**Physical activity, sedentary time, and health behaviours** are captured objectively and by self-report. Free-living physical activity PA and sedentary time will be assessed using the activPAL monitor (model activPALTM micro; PAL Technologies Ltd, Glasgow, UK), a thigh-worn tri-axial inclinometer which provides an objective measure of sitting, standing and physical activity (PA) using proprietary software, and has been found to have good measurement properties to assess sedentary, standing, and stepping time and postural transitions in adults [32]. From ActivPAL recordings, we will also derive indicators of sleep duration, sleep efficiency, sleep onset latency, nocturnal awakenings, and variability in sleep–wake patterns across days. These outcomes will complement questionnaire-based sleep measures by providing objective estimates of sleep behaviour and regularity. Participants will be asked to wear the activPAL monitor for 24-hours per day on seven consecutive days at all timepoints. A day will be considered valid if the monitor was worn for ≥ 10 hours. Participants with fewer than four valid days will be excluded from activPAL-derived analyses for that timepoint. The International PA Questionnaire Short Form (IPAQ-SF) [33] will be used to assess subjective PA levels. The IPAQ-SF includes questions about the time spent engaging in vigorous PA, moderate PA and walking in 10-min bouts or longer the past seven days. A short diet and beverages screener captures frequency of sugar-sweetened beverages/energy drinks, estimated daily caffeine intake from coffee/tea/energy drinks, fruit/vegetable intake, breakfast frequency, and late-evening eating. Alcohol Use Disorders Identification Test (AUDIT) [34] and Drug Use Disorders Identification Test (DUDIT) [35] provide risk indices for hazardous alcohol use and drug-related problems, respectively. Tobacco and snus use is recorded (status/frequency); the Fagerström Test for Nicotine Dependence [36] may be applied where dependence quantification is required.

**Somatic health and physical fitness** are evaluated by standard field measures. Anthropometry includes height (stadiometer, no shoes), weight (calibrated scale, light clothing), and waist circumference (midpoint between lowest rib and iliac crest at end-expiration). BMI (kg/m²) and waist-to-height ratio are derived, duplicate measures with re-measurement thresholds ensure reliability. Resting blood pressure is measured with a validated automated sphygmomanometer after seated rest, three readings one min apart are taken and the mean of the last two is used. Body composition is assessed by bioelectrical impedance analysis under standardized, morning, fasted conditions to improve comparability across timepoints. Cardiorespiratory fitness, or maximal oxygen uptake (VO_2max_), will be assessed through a maximum exercise test on a treadmill (Woodway, Würzburg, Germany), by physical activity professionals. A modified Balke protocol [37] will be used, where speed is held constant at 5 km ∙ h − 1 and the inclination angle increasing by one degree every minute until exhaustion within 6–12 min. Gas exchange will be continuously sampled in a mixing chamber every 30 s by having the subjects breathe into a Hans Rudolph two-way breathing valve (2700 series, Hans Rudolph Inc., Kansas City, USA). The breathing valve is connected to a Vyntus CPX (Vyaire medical, Illinois, USA), which is used to analyze the oxygen and carbon dioxide content.

**Biomarkers** index hypothesised mechanistic pathways. Salivary cortisol is used to characterise hypothalamic–pituitary–adrenal (HPA) axis dynamics via the cortisol awakening response (CAR) and diurnal slope from timed samples (awakening, + 30, + 45 min, and evening); analysis yields AUCi and slope parameters, with adherence windows prespecified. Fasting serum/plasma analytes include low-grade inflammation (high-sensitivity C-reactive protein [hs-CRP], IL-6, Tumor Necrosis Factor alpha (TNF-α)), glycaemia (glucose, HbA1c), insulin secretion/resistance, lipids (total cholesterol, HDL, LDL, triglycerides), thyroid function (TSH, free T4), prolactin, and BDNF. Samples are processed with accredited methods under a unified SOP. Pre-analytic conditions (morning, fasted; avoidance of vigorous exercise/caffeine/alcohol beforehand) are standardised and documented to reduce variability. Samples are stored at 4°C and processed within 24h, and then frozen at −80°C

### Psychological needs, motivation, and learning climate

Motivation, perceived autonomy support, and basic psychological need satisfaction will be assessed using validated self-report questionnaires based on Self-Determination Theory. The Learning Climate Questionnaire (LCQ-15) [38] will be used to assess participants’ perceptions of the extent to which the intervention environment supports autonomy. Satisfaction and frustration of the basic psychological needs for autonomy, competence, and relatedness will be assessed using the Basic Psychological Need Satisfaction and Frustration Scale (BPNSFS) [39]. The Treatment Self-Regulation Questionnaire (TSRQ) [40] will be used to assess participants’ reasons for engaging in the targeted health behaviours, including the degree to which motivation is autonomous or controlled.

**Demographics and background** (age, gender, programme and year of study, employment status and weekly hours and marital/relationship status) are collected at baseline for covariate adjustment and subgroup analyses.

Finally, **process, adherence, and safety** metrics support interpretation and implementation. Platform analytics quantify exposure and engagement (modules completed, time-on-task); scheduled check-ins document co-interventions. Adverse events are captured and graded for severity and relatedness according to the safety SOP, and post-programme acceptability/usability is assessed with a brief survey at post-programme and at 12-month follow-up (with optional qualitative interviews in a subsample) to contextualise outcomes and inform scalability within the university setting.

Unless otherwise specified, questionnaire-based outcome measures were self-administered electronically by participants under supervision of trained research staff; cognitive tasks, anthropometry, blood pressure, body composition, cardiorespiratory fitness testing, and biological sampling were administered by trained staff, and the activPAL was self-applied/worn by participants following staff instruction.

### Process evaluation

A mixed methods process evaluation will be embedded within the RCT to determine whether the programme was delivered as intended, to elucidate how and why observed effects occurred (or did not), and to inform future delivery in a university context. Guided by the MRC process-evaluation framework and Proctor’s implementation outcomes, we will assess: fidelity (adherence to content/protocol), dose delivered/received (exposure, engagement), reach/adoption (who participated), mechanisms of impact (perceived pathways and behaviour change), context (departmental timetables, exam periods, facilities), acceptability/appropriateness/feasibility, and unintended consequences (e.g., transient daytime sleepiness during sleep restriction, academic burden).

Data sources and procedures.

- Fidelity is assessed at two levels: delivery fidelity (coach adherence to the intended content, via per-session facilitator checklists) and participant engagement/dose received (session attendance, sleep-diary and homework completion, and platform/module completion).
- Engagement analytics: platform/module completion, adherence to sleep-diary
- Participant questionnaires (intervention arm): at post-programme (12 weeks) and 12-month follow-up, brief self-report items on overall experience, perceived usefulness of specific programme elements (e.g., stimulus control, sleep restriction, cognitive strategies), perceived burden, continued interaction with peers after programme end, and perceived impact on sleep, mental health, daily functioning and study performance.
- Semi-structured individual interviews will be conducted with a purposive sample of approximately 15–20 participants (sampled for sex, faculty, baseline insomnia severity, and engagement level) at post-programme and 12 months, continuing until thematic saturation is reached. Separately, 1–2 focus groups (or individual interviews, if scheduling requires) will be conducted with coaches/facilitators (target n = 4). Sessions will be audio-recorded, transcribed verbatim, and analysed thematically.
- Contamination and unintended outcomes: at each timepoint we will capture use of external sleep resources/treatments and any adverse effects; serious adverse events will be handled per the safety SOP.

Analysis and integration. Quantitative implementation indicators (fidelity scores, exposure, reach, acceptability ratings) will be summarised descriptively and related to primary/secondary outcomes. Qualitative themes will explain barriers/facilitators and hypothesised mechanisms (e.g., reduced pre-sleep arousal, strengthened circadian regularity, altered sleep cognitions). Findings will be triangulated to interpret the trial results and to refine recommendations for scaling within the university setting.

Qualitative data (interview and focus-group transcripts) will be analysed thematically in NVivo (QSR International), following Braun and Clarke’s approach to (reflexive) thematic analysis [41].

### Fieldwork staff training

Fieldwork staff training will be standardized and quality assured. We will organise a training meeting to train the fieldworkers, which will consist of students enrolled in bachelor and master in our exercise science program at the University. Standard operating procedures will describe all aspects of trial delivery including specification of equipment used in the measurement. Where a coach and participant have a pre-existing personal relationship, participants will be offered reassignment to a different group. Coaches receive practicum course credit for delivery and training.

### Procedures to maximise retention to the trial

To maximise retention at the follow-up assessments we will:

- Send students an advance reminder that follow-up measurements are upcoming, using a personalised e-mail sent two weeks ahead of the measurement dates
- Phone students one week before the scheduled postprogram and 12 month measurement sessions to arrange an appointment time for the measurements
- Send a confirmation of the date, time and location of the students appointment by e-mail
- Send a SMS in the days leading up to their appointment to remind them about the time, date and location
- Offer students who do not show up at first measurement visit a second opportunity for measurement at the University

### Research data

Data will be collected systematically during the study using electronic questionnaires, clinical assessments, and objective measurements. All data will be entered directly into a secure electronic case report form (eCRF) hosted on a USN approved platform. To ensure accuracy, data entry will follow predefined coding rules, with built-in range and completeness checks. Personal identifiers (e.g., name, e-mail) will be stored separately from research data, and all datasets used for analysis will be pseudonymised. A unique study ID will link participant information to research data.

In accordance with the USN guidelines for research data management, all data will be stored on secure servers with restricted access. Only authorised members of the research team will have access to identifiable data. Sensitive data (including personal health information) will be handled in line with GDPR and Norwegian legislation and securely archived in a controlled repository (USN’s Research Data Archive).

Data will be documented according to the FAIR principles (Findable, Accessible, Interoperable, Reusable). A README file in .txt format will be created to describe data collection methods, variables, instruments, software used, and any processing steps, together with contact information for the responsible researcher.

A Data Management Plan (DMP) has been developed and will be updated throughout the project lifecycle. The DMP describes procedures for data collection, processing, storage, archiving, and sharing. At project completion, anonymised or aggregated datasets will be archived in USN’s Research Data Archive, with appropriate access restrictions where needed. Data that can be shared will be published with an open license (e.g., Creative Commons) whenever possible, in line with USN’s policies.

Responsibility for data collection, quality control, and archiving lies with the Principal Investigator. All data handling will comply with USN’s guidelines for research data management, national regulations, and relevant ethical approvals.

### Sample size calculation

The primary outcome is change in insomnia severity as assessed by the BIS (range 0–42). In the original validation study among university students, the BIS demonstrated a standard deviation of approximately 7.4 points and excellent psychometric properties [19]. Test–retest reliability was high (r = 0.77 over two weeks), and for the present calculation we conservatively assume a correlation of r = 0.60 between baseline and follow-up scores. To date, no universally established minimal clinically important difference (MCID) exists for the BIS. Consistent with recommendations for patient-reported outcome measures [42,43] and in line with previous trials on insomnia interventions, we defined a change of 4 points (≈0.5–0.6 SD units) as clinically meaningful. This corresponds to a standardized effect size of approximately Cohen’s *d* = 0.5, i.e., a medium effect according to conventional benchmarks. Based on these parameters, a two-sided test with α = 0.05 and power = 0.90 requires 47 participants per group using an ANCOVA model. To account for an anticipated 20% attrition rate, we plan to recruit 59 participants per group, yielding a total sample size of 118. Further attrition at later stages in the study progress is accounted for by utilizing the “intention-to treat” analysis. The sample size was calculated in G*Power, version 3.1.9.7, using an ANCOVA-based formula for two independent groups with one covariate (baseline score), given the parameters above. The trial is powered for the primary outcome (BIS) only. Secondary outcomes, including cognitive, academic, mental health, physical fitness, health-behaviour and biomarker outcomes are not independently powered, and results for these outcomes will be interpreted as exploratory and hypothesis-generating, with due caution regarding multiplicity of testing.

## Statistical analysis

In accordance with the study aims, statistical analyses will be conducted to determine whether the intervention group differs from the comparison group in changes over time in primary and secondary outcomes. The key analysis will be undertaken on an intention-to-treat basis, regardless of individual engagement with the program. However, further sensitivity analyses will determine the association between attendance at intervention sessions and effectiveness (i.e., per protocol analyses). Participants referred to concomitant or external treatment during the trial remain in their originally assigned group for the intention-to-treat analysis and continue to be invited to all follow-up assessments. Receipt of concomitant treatment is recorded and will be examined in sensitivity and per-protocol analyses*.* Analysis of outcomes at each time point will be conducted using linear mixed effects regression methods (Gaussian, logistic, or other appropriate generalised linear mixed models), including fixed effects for study group and baseline outcome assessment, with participant-level random effects to account for within-subject correlation over time. Mixed-effects regression models will additionally be used to explore potential moderators and mediators of intervention effects; as the trial is not powered to detect interaction or indirect effects, these analyses will be considered exploratory and hypothesis-generatingThe patterns and extent of missing data will be examined and, if necessary, methods such as multiple imputation will be implemented to provide robust results for primary and main secondary outcomes, assuming data are missing at random.

### Health-economic evaluation

To complement the effectiveness analysis, we will conduct a trial-based cost-utility analysis using exclusively outcome measures already collected within the trial, from an intervention-provider perspective, with a 12-month time horizon matching the follow-up period. Intervention costs (coach training and salaries, materials, and coordination) will be estimated from programme records rather than participant self-report, so no additional data collection is required from participants. Health outcomes will be expressed as quality-adjusted life years (QALYs), derived from the EQ-5D-5L. We will estimate the incremental cost-effectiveness ratio (ICER), i.e., incremental cost per QALY gained, comparing the intervention with the wait-list control group. Uncertainty around cost and effect estimates will be characterised using non-parametric bootstrapping and presented as a cost-effectiveness acceptability curve. As the analysis relies solely on existing trial data, it will not capture broader societal costs such as healthcare use, medication, or productivity loss.

### Ethical considerations

The project has been approved by the Regional Committees for Medical and Health Research Ethics in Norway (reference number 950880), and the trial has been registered at ISRCTN (ISRCTN17008034). Informed written consent is a prerequisite for participation. Eligible students will receive written and oral information from project staff and have the opportunity to ask questions before consent. It will be emphasised that participation is voluntary, that participants may withdraw at any time without providing a reason, and that non-participation will not affect their studies, grades, access to student health services, or other entitlements. Students who understand the nature of the research and wish to take part will be asked to sign a paper consent form before any baseline assessments.

Focusing attention on sleep and monitoring behaviours can, in some individuals, paradoxically worsen insomnia symptoms by increasing pre-sleep arousal, sleep-related anxiety and “sleep effort”. Repeated self-monitoring (e.g., diaries, consumer trackers) may also heighten rumination or unrealistic sleep goals. In addition, behavioural components such as sleep restriction may transiently increase daytime sleepiness and reduce next-day functioning in vulnerable individuals, changes in sleep timing/intensity could exacerbate mood symptoms. To mitigate these risks, the intervention includes psychoeducation on “good-enough” sleep and normal night-to-night variability, guidance to avoid performance goals for sleep, time-limited use of sleep diaries, no real-time sleep feedback from devices, and brief mindfulness-based strategies to reduce sleep effort. Participants receive explicit safety advice (e.g., avoid driving or operating machinery when very drowsy), and time-in-bed targets can be relaxed or paused if excessive sleepiness or mood deterioration occurs. Sleep-related distress will be monitored weekly.

Data will be handled in accordance with the General Data Protection Regulation (GDPR) and institutional policies. Personally identifiable information will be stored separately from research data, analyses will use coded, de-identified datasets accessible only to authorised staff. Data will be stored on secure servers for the retention period specified in the data management plan. Participants may request deletion of identifiable data up to the point of anonymisation, subject to legal and ethical constraints.

Positive as well as negative results will be published according to the Consolidated Standards of Reporting Trials (CONSORT) guidelines. Aggregate findings will be disseminated in peer-reviewed publications and conference presentations, and a lay summary will be made available to participants.

## Discussion

This study protocol describes a pragmatic randomized controlled trial designed to evaluate the effects of a group-based sleep program for university students with insomnia. The trial addresses a clear need for scalable interventions in this population, where poor sleep is common and associated with a wide range of negative outcomes for health, mental well-being, cognitive performance, and academic attainment.

This trial is designed to test whether the programme is effective relative to no structured intervention, and whether gains are maintained over 12 months in a real-world, coach-delivered format. It is not designed to establish comparative effectiveness against brief or fully digital CBT-I formats, which represents an important direction for future research.

A major strength of the study is its pragmatic design, which enhances ecological validity and applicability in real-world university settings. By using group-based delivery, facilitated by trained coaches rather than clinical specialists, the program can be delivered at scale and with relatively modest resource demands. The program integrates established elements from CBT-I with motivational and coping frameworks, while also emphasizing peer support and active engagement. This combination has the potential to enhance adherence, internalize motivation, and sustain behaviour change over the long term. Another strength is the comprehensive measurement strategy, which spans subjective and objective sleep outcomes, mental health, cognitive and academic performance, health behaviours, and physiological indicators. The 12-month follow-up period and embedded process evaluation will provide valuable insights into both long-term effectiveness and implementation.

The trial also has limitations. As the program is not delivered by clinical therapists, elements such as cognitive therapy and sleep restriction are introduced only at a basic, educational level. While this increases feasibility, it may limit effectiveness compared to individually delivered CBT-I. Reliance on self-reported data for several outcomes introduces potential bias, although this is partly mitigated by validated instruments and objective measures (e.g., activPAL, biomarkers). Attrition is another potential concern in student populations, extensive retention strategies have therefore been built into the design. Contamination between groups cannot be completely ruled out, but the use of a wait-list control group and monitoring of co-interventions should minimize its impact. Finally, blinding of the participants is not feasible due to the nature of the behavioural sleep intervention. The lack of participant blinding may increase the risk of expectancy effects and self-report bias, as participants are aware of their treatment allocation and may consciously or unconsciously adjust their responses. This can potentially inflate observed intervention effects, particularly on subjective outcomes such as sleep quality and insomnia severity. While this limitation is inherent in most behavioural intervention trials, it underlines the importance of including objective measures where possible, as well as blinded outcome assessment and data analysis to minimize bias. Because the comparator is an inactive wait-list rather than an attention-matched control, observed effects cannot be fully attributed to the specific active ingredients of the programme as opposed to non-specific factors such as group contact, attention and expectancy. Objective outcomes (activPAL-derived sleep and activity measures, biomarkers) are less susceptible to this bias than self-report outcomes and will help contextualise the self-reported findings.

Additional considerations include the generalisability of findings, as the study population is limited to university students within a specific context, which may not fully represent young adults in other educational or cultural settings. Nevertheless, the pragmatic design enhances the external validity of the results in real-world higher education environments. Moreover, the trial will allow for exploration of potential mechanisms of change, including motivation, coping strategies, and peer support, thereby contributing to a deeper understanding of how sleep interventions exert their effects. Finally, the choice of a wait-list control balances methodological rigour with ethical considerations, ensuring that all participants ultimately receive access to the program.

Brief and fully digital CBT-I formats have shown growing efficacy (e.g., [44,45]) and are an important, low-cost option. The present trial evaluates a complementary, socially-supported group format that may particularly benefit students for whom self-guided digital approaches are insufficient. If effective, this program may represent a cost-effective and scalable approach to improving sleep and related outcomes in young adults. The results will inform not only the potential benefits of sleep interventions in higher education but also provide insight into how behaviour change programs can be implemented in non-clinical, resource-limited contexts. By addressing sleep, mental health, and performance simultaneously, the program has the potential to generate broad benefits for both students, universities and society.

## Supporting information

S1 File Study protocol 2.0. (PDF)

S2 File Completed SPIRIT checklist. (DOCX)

## Abbreviations

CBT-I: Cognitive Behavioural Therapy for Insomnia
RCT: Randomised Controlled Trial
SDT: Self-Determination Theory
SCT: Stress and Coping Theory
BIS: Bergen Insomnia Scale
ISI: Insomnia Severity Index
GSAQ: Global Sleep Assessment Questionnaire
DBAS-16: Dysfunctional Beliefs and Attitudes about Sleep
SOL: Sleep Onset Latency
PHQ-9: Patient Health Questionnaire
GAD-7: Generalized Anxiety Disorder
PSS-10: Perceived Stress Scale
WHO-5: Well-Being index
EQ-5D-5L: EuroQol 5 Dimensions – 5 Levels
IPAQ-SF: International Physical Activity Questionnaire-Short Form
AUDIT: Alcohol Use Disorders Identification Test
DUDIT: Drug Use Disorders Identification Test
BMI: Body-Mass Index
VO_2maks_: Maximal oxygen uptake
hs-CRP: High-sensitivity C-Reactive Protein
IL-6: Interleukin-6
TNF-α: Tumor Necrosis Factor alpha
HbA1c: Hemoglobin A1c
TC: Total Cholesterol
HDL: High Density Lipoprotein
LDL: Low Density Lipoprotein
TG: Triglycerides
TSH: Thyroid-Stimulating Hormone
BDNF: Brain-Derived Neurotrophic Factor
PA: Physical Activity.
