# Supplementary material for: Effects of a sleep program on sleep, performance and health in university students: Study protocol for a randomised controlled trial
Source: PLoS One. 2026 Oct 30;21(10):e0361815. doi: 10.1371/journal.pone.0361815 (PMC13722195; doi:10.1371/journal.pone.0361815)
Supplement: Supplementary file 3 [file pone.0361815.s003.docx]

The paper offers a protocol of a funded RCT evaluating the effectiveness of a group-based sleep program for university students in Norway. Students experience higher instances of poor sleep and mental health compared to those not attend university and this study offers a much-needed valuable exploration on a scalable intervention. I recommend that minor revisions to the protocol will improve the quality and offer clarity to the protocol.

-How was the intervention developed? Was there engagement with people with Lived Experience? If so at what level and what framework was followed? I think this especially important given the potential burden due to the length of the intervention and the large group format.

-Would neurodivergent students be included? How would the intervention account for their presentations and sensory experiences that may affect their following specific components of the intervention for example relating to avoid using devices before bedtime?

-Given the moderate size of the sample size it is unclear why you didn’t consider including wearables for objective sleep wake behaviours.

-Have you consider adding an acceptability measure at the end of the study rather than only collecting proxy measures of acceptability?

-What is sample size of the Qualitative interviews? Clarify whether you plan to do interviews or focus groups and what would be the sample.

-What analysis if any will be considered in relation to the biological markers? If you plan analysis, have you conducted a sample size power calculation?

-Give examples of serious adverse effects that you will consider for this population?

-As the coach trainers will also be students how you plan to address the possibility of knowing the participants? Will the trainers be compensated for their efforts including training? Will there be clinical supervision for the coaches during the intervention period?
