# Supplementary material for: Effects of a sleep program on sleep, performance and health in university students: Study protocol for a randomised controlled trial
Source: PLoS One. 2026 Oct 30;21(10):e0361815. doi: 10.1371/journal.pone.0361815 (PMC13722195; doi:10.1371/journal.pone.0361815)
Supplement: Peer review history

# Peer review: Decision Letter 0

**DOI:** 10.1371/journal.pone.0361815.r001

**Authors:** Steve Zimmerman

7 May 2026

Dear Dr. Andersen,

==============================

The manuscript has been evaluated by three reviewers, and their comments are available below and in the attached document.

The reviewers have raised a number of concerns that need attention.

-->==============================

Please submit your revised manuscript by Jun 21 2026 11:59PM. If you will need more time than this to complete your revisions, please reply to this message or contact the journal office at plosone@plos.org. When you're ready to submit your revision, log on to https://www.editorialmanager.com/pone/ and select the 'Submissions Needing Revision' folder to locate your manuscript file.

- A letter that responds to each point raised by the academic editor and reviewer(s). You should upload this letter as a separate file labeled 'Response to Reviewers'.
- A marked-up copy of your manuscript that highlights changes made to the original version. You should upload this as a separate file labeled 'Revised Manuscript with Track Changes'.
- An unmarked version of your revised paper without tracked changes. You should upload this as a separate file labeled 'Manuscript'.

If you would like to make changes to your financial disclosure, please include your updated statement in your cover letter. Guidelines for resubmitting your figure files are available below the reviewer comments at the end of this letter.

If applicable, we recommend that you deposit your laboratory protocols in protocols.io to enhance the reproducibility of your results. Protocols.io assigns your protocol its own identifier (DOI) so that it can be cited independently in the future. For instructions see: https://journals.plos.org/plosone/s/submission-guidelines#loc-laboratory-protocols. Additionally, PLOS ONE offers an option for publishing peer-reviewed Lab Protocol articles, which describe protocols hosted on protocols.io. Read more information on sharing protocols at https://plos.org/protocols?utm_medium=editorial-email&utm_source=authorletters&utm_campaign=protocols.

As the corresponding author, your ORCID iD is verified in the submission system and will appear in the published article. PLOS supports the use of ORCID, and we encourage all coauthors to register for an ORCID iD and use it as well. Please encourage your coauthors to verify their ORCID iD within the submission system before final acceptance, as unverified ORCID iDs will not appear in the published article. *Only*  the individual author can complete the verification step; PLOS staff *cannot*  verify ORCID iDs on behalf of authors.

We look forward to receiving your revised manuscript.

Kind regards,

Steve Zimmerman, PhD

Senior Editor, PLOS One

**Journal Requirements:**

1. When submitting your revision, we need you to address these additional requirements.

Please ensure that your manuscript meets PLOS ONE's style requirements, including those for file naming. The PLOS ONE style templates can be found at

https://journals.plos.org/plosone/s/file?id=wjVg/PLOSOne_formatting_sample_main_body.pdf and

https://journals.plos.org/plosone/s/file?id=ba62/PLOSOne_formatting_sample_title_authors_affiliations.pdf

2. In your cover letter, please confirm that the research you have described in your manuscript, including participant recruitment, data collection, modification, or processing, has not started and will not start until after your paper has been accepted to the journal (assuming data need to be collected or participants recruited specifically for your study). In order to proceed with your submission, you must provide confirmation.

3. When completing the data availability statement of the submission form, you indicated that you will make your data available on acceptance. We strongly recommend all authors decide on a data sharing plan before acceptance, as the process can be lengthy and hold up publication timelines. Please note that, though access restrictions are acceptable now, your entire data will need to be made freely accessible if your manuscript is accepted for publication. This policy applies to all data except where public deposition would breach compliance with the protocol approved by your research ethics board. If you are unable to adhere to our open data policy, please kindly revise your statement to explain your reasoning and we will seek the editor's input on an exemption. Please be assured that, once you have provided your new statement, the assessment of your exemption will not hold up the peer review process.

4. Your ethics statement should only appear in the Methods section of your manuscript. If your ethics statement is written in any section besides the Methods, please delete it from any other section.

5. Please include captions for your Supporting Information files at the end of your manuscript, and update any in-text citations to match accordingly. Please see our Supporting Information guidelines for more information: http://journals.plos.org/plosone/s/supporting-information.

6. If the reviewer comments include a recommendation to cite specific previously published works, please review and evaluate these publications to determine whether they are relevant and should be cited. There is no requirement to cite these works unless the editor has indicated otherwise.

[Note: HTML markup is below. Please do not edit.]

Reviewers' comments:

**Comments to the Author**

1. Does the manuscript provide a valid rationale for the proposed study, with clearly identified and justified research questions?

Reviewer #1: Partly

Reviewer #2: Yes

Reviewer #3: Yes

**********

2. Is the protocol technically sound and planned in a manner that will lead to a meaningful outcome and allow testing the stated hypotheses?

Reviewer #1: Partly

Reviewer #2: Yes

Reviewer #3: Yes

**********

3. Is the methodology feasible and described in sufficient detail to allow the work to be replicable?

Reviewer #1: No

Reviewer #2: Yes

Reviewer #3: Yes

**********

4. Have the authors described where all data underlying the findings will be made available when the study is complete?

The PLOS Data policy

Reviewer #1: Yes

Reviewer #2: No

Reviewer #3: Yes

**********

5. Is the manuscript presented in an intelligible fashion and written in standard English?

*PLOS ONE*

Reviewer #1: Yes

Reviewer #2: Yes

Reviewer #3: Yes

**********

Please use the space provided to explain your answers to the questions above and, if applicable, provide comments about issues authors must address before this protocol can be accepted for publication. You may also include additional comments for the author, including concerns about research or publication ethics.

You may also provide optional suggestions and comments to authors that they might find helpful in planning their study.

**Reviewer #1:**  The manuscript requires improvement.

Line 122: Typo ‘whit’

Line 132: Is English required for this study? It was not clearly stated whether the Scandinavian or English versions of the questionnaires were used. If participants are required to communicate in a Scandinavian language (e.g., Norwegian), the questionnaires should preferably be administered in the corresponding Scandinavian version to ensure linguistic and cultural appropriateness.

References for all assessment tools and questionnaires mentioned should be provided, including details of validation studies where applicable, or, at a minimum, a statement confirming that all instruments have been validated in the language version used.

Line 248: The details of sample size determination are to be provided.

Line 304: The sentence is to be improved. It depends on the type/pattern of missing data.

Line 354, 359, 379 etc: [REF]?

A statement ‘All outcome measures were collected using self-administered questionnaires completed by the participants.’ is to be provided.

Line 497:The formula or software used to derive the sample size is to be stated.

The statistical software, the publisher's name, and its version, as well as the accepted level of significance, are to be stated.

Line 503: The section is to be named ‘Statistical analyses’

For qualitative analysis, the software and analysis approach are to be provided.

Line 519: The reference number is to be stated.

Line 539: Full name for GDPR is to be stated.

Line 548 & Line 229-230: The statement ‘The wait-list control group will be offered access to the programme after completing the 12-month follow-up assessments’ is repeated.

Line 509-512: The sentence could be improved e.g. Analyses of outcomes at each time point will be conducted using linear mixed-effects regression models (Gaussian, logistic, or other appropriate generalised linear mixed models), including fixed effects for study group and baseline outcome assessment, with participant-level random effects to account for within-subject correlation over time.

Line 512-513: Sentence could be improved e.g. Mixed-effects regression models will be used to examine potential moderators and mediators of the intervention effects.

The sample size was calculated using ANCOVA. Moderator and mediator analyses were conducted using regression models and are to be interpreted as exploratory, as the study is not specifically powered to detect interaction or indirect effects.

**Reviewer #2:**  Re- sleep programs on sleep, performance and health in university students – RCT protocol

This is a protocol paper on a pragmatic 2-arm RCT for improving sleep, performance and health of the university students. The youth sleep problems including university students and insomnia are major health problems that will need further systematic intervention, especially at pragmatic implementation. From this implementation angle, further pragmatic study is needed. However, there are a number of major issues that the authors may need to rethink and reconceptualize the pragmatic trial design.

1. The primary outcome of the study is whether this 12 weeks 90 min sessions + 9 monthly meetings with a combination of CBT-I (theory and strategies), stress coping and motivational approach, guided by a trained coach will improve the insomnia severity as primary outcome, and a list of other secondary outcomes including cognitive and academic, mental health and healthy behaviors, physical fitness and somatic health, and a potential series of immune and cortisol markers. The comparison group is the wait-list control.

a. As CBT-I, in various formats, face-to-face (individual and group), internet (guided/self), telephone, app-based (guided, fully automatic) has been shown to improve insomnia outcome consistently, what will be this additional whole year intervention (12 weeks + 9 monthly follow-up meeting) will add on the data, especially in comparing to an inactive control (waitlist group)?

b. This 12 week mixed method interventions with a trained coach – with both groups and individual add on session, followed by 9 monthly ‘light-touch’ session will indeed be an intense resources and demand to the young university students. As the study has mentioned the cost-effectiveness and scalability of intervention, has the study planned any cost-effectiveness measures? In terms of scalability, how will the study translate this intense demand 12 weekly (90 mins/session) + 9 monthly sessions into a scalable intervention, given the fact that most CBT-I will work for 4-8 sessions and recent digital CBT-I intervention with only a few weeks intervention will help to improve most insomnia outcomes (Hwang et al NPJ DM 2025) and recent digital app-based CBT-I intervention could improve depression and prevent depressive disorder (Chen et al Plos Med 2025). Thus, the current study design will need to take reference from the latest evidences of the digital intervention for potentially scalable and cost-effective measures.

c. Wait-list control group – as the CBT-I has been shown to be well established effective measure, why the wait-list control group was used? The most problematic is the interpretation – if the results were positive, was it simply due to non-specific intervention/attention effect, rather than from the active interventional component?

d. Coach vs therapist – the study design has the strength of using coach, instead of therapists, but did they aim to compare the cost-effectiveness of coach led vs therapist led RCT?

e. Duration and augmented FU – the duration of the intervention is quite long - 12 weeks, and there are 9 additional monthly FU with therapeutic element (eg response prevention). What is the rationale of developing such long intense intervention, if the study is planning for a pragmatic trial?

2. Sample size – the estimation of sample size was based on traditional effect size of the CBT-I intervention. A sample size of 59 each arm is not ‘large’ in any pragmatic trial sense (pg 5, ln 92).

3. Other outcomes measures – the strength (or potential problems) of the study design is to investigate a comprehensive holistic measure of the mental, physical, exercise, objective sleep, and biological markers. However, will this sample size be able to address these comprehensive measures?

4. Randomization – the potential contamination issue has been considered by the authors, but it has been regarded to be minimal. However, more evidence is needed to regard this contamination issue as minimal repercussion. In the reference quoted (Torgeson DL. BMI 2001), increase in sample size was recommended to tackle the contamination issue.

5. Insomnia target – specific health issues that could explain insomnia will be excluded. However, it is well established that CBT-I is an effective treatment for comorbid insomnia (Hertenstein et al Sleep med rev 2022). Why they have to be excluded? How about drug abuse and alcohol screening?

6. Fidelity measures- what are the measures? For the session completion? Or more for behavioral change, wearable adherences…

7. Other concomitant interventions – it was unclear if the subjects were to be referred out, will the study regard them as dropout/completion?

Others

a. Some of the ‘references’ in the text were not quoted (REF)

b. Some minor typo – eg whit = with

c. The detailed data management plan and, ethics consideration has to be commended.

**Reviewer #3:**  The paper offers a protocol of a funded RCT evaluating the effectiveness of a group-based sleep program for university students in Norway. Students experience higher instances of poor sleep and mental health compared to those not attend university and this study offers a much-needed valuable exploration on a scalable intervention. I recommend that minor revisions to the protocol will improve the quality and offer clarity to the protocol.

**********

what does this mean?). If published, this will include your full peer review and any attached files.

If you choose “no”, your identity will remain anonymous but your review may still be made public.

**Do you want your identity to be public for this peer review?**  For information about this choice, including consent withdrawal, please see our Privacy Policy

Reviewer #1: No

Reviewer #2: No

Reviewer #3: **Yes:**  Maria Gardani

**********

[NOTE: If reviewer comments were submitted as an attachment file, they will be attached to this email and accessible via the submission site. Please log into your account, locate the manuscript record, and check for the action link "View Attachments". If this link does not appear, there are no attachment files.]

To ensure your figures meet our technical requirements, please review our figure guidelines: https://journals.plos.org/plosone/s/figures

You may also use PLOS’s free figure tool, NAAS, to help you prepare publication quality figures: https://journals.plos.org/plosone/s/figures#loc-tools-for-figure-preparation.

NAAS will assess whether your figures meet our technical requirements by comparing each figure against our figure specifications.

Attachment Submitted filename: PONE-D-26-01232.docx

# Author comment: Author response to Decision Letter 1

**DOI:** 10.1371/journal.pone.0361815.r002

3 Sep 2026

Point by point reply to reviewers

Reviewer #1: The manuscript requires improvement.

Line 122: Typo ‘whit’

Response: Corrected.

Line 132: Is English required for this study? It was not clearly stated whether the Scandinavian or English versions of the questionnaires were used. If participants are required to communicate in a Scandinavian language (e.g., Norwegian), the questionnaires should preferably be administered in the corresponding Scandinavian version to ensure linguistic and cultural appropriateness.

Response: Agreed, this was not stated. English is not required (inclusion criterion is "understand and speak a Scandinavian language"); validated and/or officially translated Norwegian-language versions exist for all questionnaires and outcome measures used in this trial, and these versions will be used. We have added a statement to this in the ms.

References for all assessment tools and questionnaires mentioned should be provided, including details of validation studies where applicable, or, at a minimum, a statement confirming that all instruments have been validated in the language version used.

Response: We agree and have added citations throughout the manuscript for all assessment tools and questionnaires, at each point where they are mentioned in the text. Validated and/or officially translated Norwegian-language versions exist for these instruments, and these are the versions used in the trial (see also our response to the comment on Line 132, above).

Line 248: The details of sample size determination are to be provided.

Response: Addressed together with the comment on line 497 below (formula/software for the sample size calculation), since both point to the same section.

Line 304: The sentence is to be improved. It depends on the type/pattern of missing data.

Response: Agreed. The original sentence asserted multiple imputation without qualification. The fuller, correctly-qualified statement already appears later under the analysis plan. We have shortened the Procedures-section sentence to avoid an unqualified duplicate claim and cross-referenced the analysis section.

Line 354, 359, 379 etc: [REF]?

Response: References have been inserted.

A statement ‘All outcome measures were collected using self-administered questionnaires completed by the participants.’ is to be provided.

Response: Added, with the necessary caveat that not all outcomes are self-administered questionnaires (several are objective/interviewer-administered).

Line 497: The formula or software used to derive the sample size is to be stated.

The statistical software, the publisher's name, and its version, as well as the accepted level of significance, are to be stated.

Response: We have added the requested detail. We also take this opportunity to state explicitly that the sample size was calculated for the Bergen Insomnia Scale (BIS) specifically.

Line 503: The section is to be named ‘Statistical analyses’

Response: renamed as requested.

For qualitative analysis, the software and analysis approach are to be provided.

Response: Added.

Line 519: The reference number is to be stated.

Response: Added.

Line 539: Full name for GDPR is to be stated.

Response: Added at first use.

Line 548 & Line 229-230: The statement ‘The wait-list control group will be offered access to the programme after completing the 12-month follow-up assessments’ is repeated.

Response: Agreed; the sentence in the Ethical considerations section duplicated the fuller statement already given under Comparison group. We have removed the duplicate.

Line 509-512: The sentence could be improved e.g. Analyses of outcomes at each time point will be conducted using linear mixed-effects regression models (Gaussian, logistic, or other appropriate generalised linear mixed models), including fixed effects for study group and baseline outcome assessment, with participant-level random effects to account for within-subject correlation over time.

Response: We are grateful for the suggested wording and have adopted it directly.

Line 512-513: Sentence could be improved e.g. Mixed-effects regression models will be used to examine potential moderators and mediators of the intervention effects.

The sample size was calculated using ANCOVA. Moderator and mediator analyses were conducted using regression models and are to be interpreted as exploratory, as the study is not specifically powered to detect interaction or indirect effects.

Response: Agreed and adopted, combining the reviewer’s suggested wording with the explicit exploratory framing requested.

Reviewer #2: Re- sleep programs on sleep, performance and health in university students – RCT protocol

This is a protocol paper on a pragmatic 2-arm RCT for improving sleep, performance and health of the university students. The youth sleep problems including university students and insomnia are major health problems that will need further systematic intervention, especially at pragmatic implementation. From this implementation angle, further pragmatic study is needed. However, there are a number of major issues that the authors may need to rethink and reconceptualize the pragmatic trial design.

1. The primary outcome of the study is whether this 12 weeks 90 min sessions + 9 monthly meetings with a combination of CBT-I (theory and strategies), stress coping and motivational approach, guided by a trained coach will improve the insomnia severity as primary outcome, and a list of other secondary outcomes including cognitive and academic, mental health and healthy behaviors, physical fitness and somatic health, and a potential series of immune and cortisol markers. The comparison group is the wait-list control.

a. As CBT-I, in various formats, face-to-face (individual and group), internet (guided/self), telephone, app-based (guided, fully automatic) has been shown to improve insomnia outcome consistently, what will be this additional whole year intervention (12 weeks + 9 monthly follow-up meeting) will add on the data, especially in comparing to an inactive control (waitlist group)?

Response: This is a fair question and we have expanded the rationale in the Discussion. Existing CBT-I formats (including brief and digital variants) are typically evaluated over short follow-up windows and in samples with high baseline motivation/compliance, evidence on whether gains are sustained over a full academic year, in a pragmatic, coach-delivered, group-based format embedded in a real university setting, is much more limited. The extended monthly follow-up (rather than the core 12-week programme alone) is specifically designed to test whether continued, lower-intensity contact improves long-term maintenance and relapse prevention, a distinct question from short-term efficacy, which is already reasonably well established. A head-to-head comparison against brief/digital CBT-I would answer a different (also valuable) comparative-effectiveness question, and would require a differently powered, multi-arm design; we have added a sentence flagging this as an important next step rather than something this trial is designed to answer. Lastly, the control group is not completely inactive, they receive sleep hygiene advice.

b. This 12 week mixed method interventions with a trained coach – with both groups and individual add on session, followed by 9 monthly ‘light-touch’ session will indeed be an intense resources and demand to the young university students. As the study has mentioned the cost-effectiveness and scalability of intervention, has the study planned any cost-effectiveness measures? In terms of scalability, how will the study translate this intense demand 12 weekly (90 mins/session) + 9 monthly sessions into a scalable intervention, given the fact that most CBT-I will work for 4-8 sessions and recent digital CBT-I intervention with only a few weeks intervention will help to improve most insomnia outcomes (Hwang et al NPJ DM 2025) and recent digital app-based CBT-I intervention could improve depression and prevent depressive disorder (Chen et al Plos Med 2025). Thus, the current study design will need to take reference from the latest evidences of the digital intervention for potentially scalable and cost-effective measures.

Response: We have reviewed both papers. Hwang et al. (2025) is a meta-analysis of 29 RCTs of fully automated digital CBT-I, showing moderate-to-large effects on insomnia severity that were nonetheless smaller than therapist-assisted formats. Chen et al. (2025) is a randomised trial of a 6-week app-based CBT-I in youth with insomnia and subclinical depression, showing a reduced 12-month incidence of major depressive disorder. Both are relevant and we now cite them in the discussion as evidence that brief/digital formats have real, growing efficacy, while also noting that fully automated formats show smaller effects than supported formats, and that our trial tests a complementary, socially-supported group format that may particularly suit students for whom self-guided digital tools have not worked, or who value in-person peer contact (a known protective factor against the loneliness and mental-health burden documented in this population, e.g. in the national SHoT survey). On cost-effectiveness, yes we will conduct this and a brief section has been added to the ms: please see line 533 “health-economic evaluation”

c. Wait-list control group – as the CBT-I has been shown to be well established effective measure, why the wait-list control group was used? The most problematic is the interpretation – if the results were positive, was it simply due to non-specific intervention/attention effect, rather than from the active interventional component?

Response: We agree this is an important limitation and have made it more explicit. A wait-list is a recognised, ethically favourable design for a first pragmatic effectiveness test (all participants ultimately receive the programme; resource constraints of an academic pragmatic trial make a matched attention-control arm infeasible), but it cannot separate specific CBT-I content effects from non-specific effects of group contact, attention and expectancy. We have expanded the Limitations paragraph to state this explicitly rather than only alluding to blinding.

d. Coach vs therapist – the study design has the strength of using coach, instead of therapists, but did they aim to compare the cost-effectiveness of coach led vs therapist led RCT?

Response: A therapist-led comparator arm would require a third trial arm and a substantially larger sample. The existing literature already establishes efficacy of therapist-led CBT-I. This trial’s contribution is instead to test feasibility and effectiveness of a task-shifted, coach-led delivery model at scale, which is a distinct and, we would argue, equally important question given workforce and access constraints in student mental-health services.

e. Duration and augmented FU – the duration of the intervention is quite long - 12 weeks, and there are 9 additional monthly FU with therapeutic element (eg response prevention). What is the rationale of developing such long intense intervention, if the study is planning for a pragmatic trial?

Response: We agree the term "pragmatic" could be misread here. In trial methodology, "pragmatic" (e.g. the PRECIS-2 framework) describes the trial’s conditions – real-world setting, non-specialist delivery personnel, broad eligibility, intention-to-treat analysis – rather than the intensity or brevity of the intervention itself; a pragmatic trial can test an intensive intervention delivered under everyday conditions. We have added a clarifying sentence, and re-stated the rationale for the extended monthly follow-up specifically (testing durability/relapse prevention, as noted under Comment 1a).

2. Sample size – the estimation of sample size was based on traditional effect size of the CBT-I intervention. A sample size of 59 each arm is not ‘large’ in any pragmatic trial sense (pg 5, ln 92).

Response: Agreed – on reflection, "large" at line 92 was intended to describe the comprehensiveness of the intervention and outcome battery and the scale of intended delivery within the university, not the sample size, but we see how it reads as a claim about statistical power. We have removed the word to avoid this impression.

3. Other outcomes measures – the strength (or potential problems) of the study design is to investigate a comprehensive holistic measure of the mental, physical, exercise, objective sleep, and biological markers. However, will this sample size be able to address these comprehensive measures?

Response: No, and the manuscript did not previously say so explicitly. The sample size (N=118) was calculated for the primary outcome only (BIS; see Sample size calculation, which uses BIS alone). We have made this explicit and stated plainly that secondary outcomes, including cognitive, mental health, fitness and biomarker outcomes, are not independently powered and should be interpreted as exploratory. This also resolves an internal inconsistency worth flagging: the manuscript currently describes both BIS and ISI together as "the primary outcome" in the Abstract and Methods (lines 40–41, 307, 315–317), while the power calculation is based on BIS alone. We recommend explicitly designating BIS as the primary outcome (consistent with the power calculation) and ISI as a key secondary/confirmatory sleep outcome, rather than leaving two co-primary outcomes without a stated multiplicity correction.

4. Randomization – the potential contamination issue has been considered by the authors, but it has been regarded to be minimal. However, more evidence is needed to regard this contamination issue as minimal repercussion. In the reference quoted (Torgeson DL. BMI 2001), increase in sample size was recommended to tackle the contamination issue.

Response: We have strengthened the justification. Torgerson (2001) does note that meaningful contamination risk favours either cluster randomisation or a larger individually-randomised sample, but also concludes that individual randomisation remains appropriate when contamination is genuinely unlikely to be substantial – which we believe is the case for this intervention: it is an intensive, in-person, group- and coach-delivered programme with a workbook, not a brief, easily-shared resource like a leaflet or app, making informal "transfer" of the active intervention from an intervention-arm participant to a wait-listed participant unlikely. We have also brought forward, into the Randomisation section itself, the contamination-monitoring plan that was previously described only later under Process evaluation, and added a pre-specified sensitivity analysis.

5. Insomnia target – specific health issues that could explain insomnia will be excluded. However, it is well established that CBT-I is an effective treatment for comorbid insomnia (Hertenstein et al Sleep med rev 2022). Why they have to be excluded? How about drug abuse and alcohol screening?

Response: We have reviewed Hertenstein et al. (2022), a systematic review and meta-analysis showing CBT-I is effective for insomnia comorbid with psychiatric disorders. This is consistent with our design rather than in tension with it: our exclusion criteria target only moderate-to-severe comorbid conditions ("moderate or severe health issues related to their insomnia diagnosis", "moderate or severe mental illness", "unstable or severe medical/neurological conditions"), not comorbidity in general – mild-to-moderate anxiety or depressive symptoms, for example, are not grounds for exclusion. The rationale is one of safety and scope of practice: the programme is delivered by trained (non-clinical) student coaches rather than clinicians, so participants whose needs exceed what a coach-led group format can safely support are referred to professional services instead (as already stated at line 191) rather than enrolled. On substance use: AUDIT and DUDIT are already administered as outcome/covariate measures (Table 2) rather than as an a priori exclusion criterion; we have added a sentence clarifying what happens if a participant screens positive for hazardous use.

6. Fidelity measures- what are the measures? For the session completion? Or more for behavioral change, wearable adherences…

Response: The protocol already distinguishes two constructs but this was not made explicit enough. We have clarified the distinction between (i) delivery fidelity – whether coaches delivered the intended content, assessed via facilitator checklists – and (ii) participant engagement/dose received – attendance, homework and sleep-diary completion, and platform/module completion. We have also added an explicit wear-time validity criterion for the activPAL data, which was previously undefined.

7. Other concomitant interventions – it was unclear if the subjects were to be referred out, will the study regard them as dropout/completion?

Response: Clarified, consistent with the trial’s stated intention-to-treat approach: participants referred to external/concomitant treatment remain in their originally assigned group for the primary intention-to-treat analysis and continue to be invited to all follow-up assessments; receipt of concomitant treatment is recorded as a co-intervention (as already described for contamination monitoring) and will be examined in per-protocol and sensitivity analyses.

Others

a. Some of the ‘references’ in the text were not quoted (REF)

Response: inserted

b. Some minor typo – eg whit = with

Response: Corrected

c. The detailed data management plan and, ethics consideration has to be commended.

Response: We thank the reviewer for this positive comment

Reviewer #3: The paper offers a protocol of a funded RCT evaluating the effectiveness of a group-based sleep program for university students in Norway. Students experience higher instances of poor sleep and mental health compared to those not attend university and this study offers a much-needed valuable exploration on a scalable intervention. I recommend that minor revisions to the protocol will improve the quality and offer clarity to the protocol.

-How was the intervention developed? Was there engagement with people with Lived Experience? If so at what level and what framework was followed? I think this especially important given the potential burden due to the length of the intervention and the large group format.

Response: The programme was developed by the research team, grounded in established CBT-I, Self-Determination Theory and Stress and Coping Theory frameworks. In direct response to this comment, we have also specified that, prior to the full trial, the programme content and session structure will be piloted with a group of students and refined based on their feedback; we have added this to the end of the Intervention program section.

-Would neurodivergent students be included? How would the intervention account for their presentations and sensory experiences that may affect their following specific components of the intervention for example relating to avoid using devices before bedtime?

Response: The trial does not exclude neurodivergent students who do not otherwise meet the exclusion criteria above. However, the standardised programme content has not been formally adapted for neurodivergent presentations (e.g., sensory sensitivities, or difficulty with blanket recommendations such as avoiding devices before bedtime). Coaches will be trained to individualise specific behavioural recommendations where a strict version is not feasible for a given participant (e.g., night-mode/blue-light filtering as an alternative where full device avoidance is impractical), while preserving the core CBT-I components.

-Given the moderate size of the sample size it is unclear why you didn’t consider including wearables for objective sleep wake behaviours.

Response: We agree that wearables could have provided useful complementary information. However, insomnia is primarily defined by subjective sleep difficulties and daytime impairment, and current guidelines do not require actigraphy for routine insomnia assessment. In addition, wearables may misclassify quiet wakefulness as sleep, which is particularly relevant in insomnia. We therefore prioritised validated questionnaires and sleep diaries that were closely aligned with the study aims, while acknowledging the absence of objective sleep measures as a limitation. That said, we will use data from the ActivPAL to look at sleep.

-Have you consider adding an acceptability measure at the end of the study rather than only collecting proxy measures of acceptability?

Response: Agreed. Acceptability/usability is currently assessed only at post-programme (12 weeks); we have added the same measure at the 12-month follow-up, so that perceived value can also be assessed once participants have had time to judge whether changes were sustained, which also strengthens the scalability argument made in the Discussion.

-What is sample size of the Qualitative interviews? Clarify whether you plan to do interviews or focus groups and what would be the sample.

Response: Clarified. The original wording ("interviews/focus groups… and/or… focus groups or interviews") left the design underspecified; we have separated the participant and coach/facilitator strands and specified target numbers.

-What analysis if any will be considered in relation to the biological markers? If you plan analysis, have you conducted a sample size power calculation?

Response: As clarified in our response to Reviewer #2, Comment 3, no separate power calculation was conducted for biomarker outcomes; the trial is powered for the primary sleep outcome only. Biomarker outcomes will be analysed using the same linear mixed-effects framework as other secondary outcomes and interpreted as exploratory. Main purpose is to see if improved sleep effects biomarkers.

-Give examples of serious adverse effects that you will consider for this population?

Response: Noting more than already written in the protocol: “Focusing attention on sleep and monitoring behaviours can, in some individuals, paradoxically worsen insomnia symptoms by increasing pre-sleep arousal, sleep-related anxiety and “sleep effort”. Repeated self-monitoring (e.g., diaries, consumer trackers) may also heighten rumination or unrealistic sleep goals. In addition, behavioural components such as sleep restriction may transiently increase daytime sleepiness and reduce next-day functioning in vulnerable individuals, changes in sleep timing/intensity could exacerbate mood symptoms. To mitigate these risks, the intervention includes psychoeducation on “good-enough” sleep and normal night-to-night variability, guidance to avoid performance goals for sleep, time-limited use of sleep diaries, no real-time sleep feedback from devices, and brief mindfulness-based strategies to reduce sleep effort. Participants receive explicit safety advice (e.g., avoid driving or operating machinery when very drowsy), and time-in-bed targets can be relaxed or paused if excessive sleepiness or mood deterioration occurs. Sleep-related distress will be monitored weekly.»

-As the coach trainers will also be students how you plan to address the possibility of knowing the participants? Will the trainers be compensated for their efforts including training? Will there be clinical supervision for the coaches during the intervention period?

Response: we have added the following text under “Fieldwork staff training”: “Where a coach and participant have a pre-existing personal relationship, participants will be offered reassignment to a different group. Coaches receive practicum course credit for delivery and training”. To the last part of your question this text follows as an Asterix to the “intervention program”: “The sessions provide participants with tools for self-reflection and peer discussion. Coaches will be trained to facilitate the exercises but will not act as therapists. Participants who require more comprehensive therapy will be encouraged to seek professional health services.”

Attachment Submitted filename: Response to Reviewers.docx

# Peer review: Decision Letter 1

**DOI:** 10.1371/journal.pone.0361815.r003

**Authors:** Xian-Liang Liu

24 Sep 2026

Effects of a sleep program on sleep, performance and health in university students: study protocol for a randomised controlled trial

PONE-D-26-01232R1

Dear Dr. Andersen,

We’re pleased to inform you that your manuscript has been judged scientifically suitable for publication and will be formally accepted for publication once it meets all outstanding technical requirements.

Within one week, you’ll receive an e-mail detailing the required amendments. When these have been addressed, you’ll receive a formal acceptance letter and your manuscript will be scheduled for publication.

An invoice will be generated when your article is formally accepted. Please note, if your institution has a publishing partnership with PLOS and your article meets the relevant criteria, all or part of your publication costs will be covered. Please make sure your user information is up-to-date by logging into Editorial Manager at Editorial Manager® and clicking the ‘Update My Information' link at the top of the page. For questions related to billing, please contact billing support.

If your institution or institutions have a press office, please notify them about your upcoming paper to help maximize its impact. If they’ll be preparing press materials, please inform our press team as soon as possible -- no later than 48 hours after receiving the formal acceptance. Your manuscript will remain under strict press embargo until 2 pm Eastern Time on the date of publication. For more information, please contact onepress@plos.org.

Kind regards,

Xian-Liang Liu

Academic Editor

PLOS One

Additional Editor Comments (optional):

Reviewers' comments:

Reviewer's Responses to Questions

**Comments to the Author**

1. Does the manuscript provide a valid rationale for the proposed study, with clearly identified and justified research questions?

Reviewer #1: Partly

Reviewer #3: Yes

**********

2. Is the protocol technically sound and planned in a manner that will lead to a meaningful outcome and allow testing the stated hypotheses?

Reviewer #1: Partly

Reviewer #3: Yes

**********

3. Is the methodology feasible and described in sufficient detail to allow the work to be replicable?

Reviewer #1: Yes

Reviewer #3: Yes

**********

4. Have the authors described where all data underlying the findings will be made available when the study is complete?

The PLOS Data policy

Reviewer #1: Yes

Reviewer #3: Yes

**********

5. Is the manuscript presented in an intelligible fashion and written in standard English?

*PLOS ONE*

Reviewer #1: Yes

Reviewer #3: Yes

**********

Please use the space provided to explain your answers to the questions above and, if applicable, provide comments about issues authors must address before this protocol can be accepted for publication. You may also include additional comments for the author, including concerns about research or publication ethics.

You may also provide optional suggestions and comments to authors that they might find helpful in planning their study.

Reviewer #1: The authors have adequately addressed the comments raised by the reviewer(s) and have made the necessary revisions to the manuscript. I have no further comments.

Reviewer #3: The authors responded or revised the manuscript following the suggestions. I recommend acceptance and looking forward to hear about their findings.

**********

what does this mean?). If published, this will include your full peer review and any attached files.

If you choose “no”, your identity will remain anonymous but your review may still be made public.

**Do you want your identity to be public for this peer review?**  For information about this choice, including consent withdrawal, please see our Privacy Policy

Reviewer #1: No

Reviewer #3: No

**********

# Editor report: Acceptance letter

**DOI:** 10.1371/journal.pone.0361815.r004

**Authors:** Xian-Liang Liu

PONE-D-26-01232R1

PLOS One

Dear Dr. Andersen,

I'm pleased to inform you that your manuscript has been deemed suitable for publication in PLOS One. Congratulations! Your manuscript is now being handed over to our production team.

At this stage, our production department will prepare your paper for publication. This includes ensuring the following:

* All references, tables, and figures are properly cited

* All relevant supporting information is included in the manuscript submission,

* There are no issues that prevent the paper from being properly typeset

You will receive further instructions from the production team, including instructions on how to review your proof when it is ready. Please keep in mind that we are working through a large volume of accepted articles, so please give us a few days to review your paper and let you know the next and final steps.

Lastly, if your institution or institutions have a press office, please let them know about your upcoming paper now to help maximize its impact. If they'll be preparing press materials, please inform our press team within the next 48 hours. Your manuscript will remain under strict press embargo until 2 pm Eastern Time on the date of publication. For more information, please contact onepress@plos.org.

You will receive an invoice from PLOS for your publication fee after your manuscript has reached the completed accept phase. If you receive an email requesting payment before acceptance or for any other service, this may be a phishing scheme. Learn how to identify phishing emails and protect your accounts at https://explore.plos.org/phishing.

If we can help with anything else, please email us at customercare@plos.org.

Thank you for submitting your work to PLOS One and supporting open access.

Kind regards,

PLOS One Editorial Office Staff

on behalf of

Dr. Xian-Liang Liu

Academic Editor

PLOS One
